# Hooking the scientific community on thorny-headed worms: interesting and exciting facts, knowledge gaps and perspectives for research directions on Acanthocephala[Fn FN1]

**DOI:** 10.1051/parasite/2023026

**Published:** 2023-06-22

**Authors:** Marie-Jeanne Perrot-Minnot, Camille-Sophie Cozzarolo, Omar Amin, Daniel Barčák, Alexandre Bauer, Vlatka Filipović Marijić, Martín García-Varela, Jesús Servando Hernández-Orts, T.T. Yen Le, Milen Nachev, Martina Orosová, Thierry Rigaud, Sara Šariri, Rémi Wattier, Florian Reyda, Bernd Sures

**Affiliations:** 1 Biogéosciences, UMR 6282 CNRS, Université de Bourgogne 6 Boulevard Gabriel 21000 Dijon France; 2 Parasitology Center, Inc. (PCI) and Institute of Parasitic Diseases (IPD) 11445 E. Via Linda 2-419 Scottsdale AZ 85259 United States; 3 Institute of Parasitology, Slovak Academy of Sciences Hlinkova 3 040 01 Košice Slovak Republic; 4 Ruđer Bošković Institute Bijenička cesta 54 10000 Zagreb Croatia; 5 Departamento de Zoología, Instituto de Biología, Universidad Nacional Autónoma de México Ciudad de México 04510 México; 6 Institute of Parasitology, Biology Centre, Czech Academy of Sciences Branišovská 1160/31 370 05 České Budějovice Czech Republic; 7 Natural History Museum, London Cromwell Road London SW7 5BD United Kingdom; 8 Aquatic Ecology and Centre for Water and Environmental Research, University of Duisburg-Essen Universitaetsstr. 5 45141 Essen Germany; 9 Biology Department and Biological Field Station, State University of New York at Oneonta Ravine Parkway Oneonta NY 13820 United States; 10 Research Center One Health Ruhr, Research Alliance Ruhr, University of Duisburg-Essen Essen Germany

**Keywords:** Acanthocephala, Environmental parasitology, Host ranges, Integrative taxonomy, Transmission strategies

## Abstract

Although interest in Acanthocephala seems to have reached only a small community of researchers worldwide, we show in this opinion article that this group of parasites is composed of excellent model organisms for studying key questions in parasite molecular biology and cytogenetics, evolutionary ecology, and ecotoxicology. Their shared ancestry with free-living rotifers makes them an ideal group to explore the origins of the parasitic lifestyle and evolutionary drivers of host shifts and environmental transitions. They also provide useful features in the quest to decipher the proximate mechanisms of parasite-induced phenotypic alterations and better understand the evolution of behavioral manipulation. From an applied perspective, acanthocephalans’ ability to accumulate contaminants offers useful opportunities to monitor the impacts – and evaluate the possible mitigation – of anthropogenic pollutants on aquatic fauna and develop the environmental parasitology framework. However, exploring these exciting research avenues will require connecting fragmentary knowledge by enlarging the taxonomic coverage of molecular and phenotypic data. In this opinion paper, we highlight the needs and opportunities of research on Acanthocephala in three main directions: (i) integrative taxonomy (including non-molecular tools) and phylogeny-based comparative analysis; (ii) ecology and evolution of life cycles, transmission strategies and host ranges; and (iii) environmental issues related to global changes, including ecotoxicology. In each section, the most promising ideas and developments are presented based on selected case studies, with the goal that the present and future generations of parasitologists further explore and increase knowledge of Acanthocephala.

## Abstract (fr)

Bien que l’intérêt pour les acanthocéphales semble n’avoir atteint qu’un petit nombre de chercheurs dans le monde, nous montrons dans cet article que ce groupe de parasites est composé d’excellents organismes modèles pour étudier les questions en suspens en biologie moléculaire et cytogénétique, écologie évolutive et écotoxicologie. Leur ascendance partagée avec les rotifères en fait un groupe idéal pour explorer les origines du mode de vie parasitaire et les moteurs évolutifs des changements d’hôtes et des transitions environnementales. Ils présentent également des caractéristiques intéressantes pour l’étude des mécanismes proximaux sous-tendant les altérations phénotypiques induites par les parasites, et ainsi mieux comprendre l’évolution de la manipulation comportementale. D’un point de vue appliqué, la capacité des acanthocéphales à accumuler les contaminants offre des opportunités utiles pour surveiller les impacts - et évaluer les possibilités d’atténuation - des pollutions anthropiques sur la faune aquatique et développer le domaine de la parasitologie environnementale. Cependant, l’exploration de ces pistes de recherche passionnantes nécessitera de relier des connaissances fragmentaires en élargissant la couverture taxonomique des données moléculaires et phénotypiques. Dans cet article, nous présentons l’état actuel de la recherche sur les acanthocéphales selon trois axes principaux : (i) la taxonomie intégrative (y compris les outils non-moléculaires) et la phylogénie à des fins d’analyse comparative ; (ii) l’écologie et l’évolution des cycles de vie, des stratégies d’exploitation des hôtes et de transmission ; (iii) les questions environnementales liées aux changements globaux, y compris l’écotoxicologie. Dans chaque section, nous soulignons les besoins et les opportunités, en espérant que cela incitera une nouvelle génération de parasitologues à s’intéresser aux acanthocéphales.

## Introduction: acanthocephalan origins and article outline

**Figure 1:**
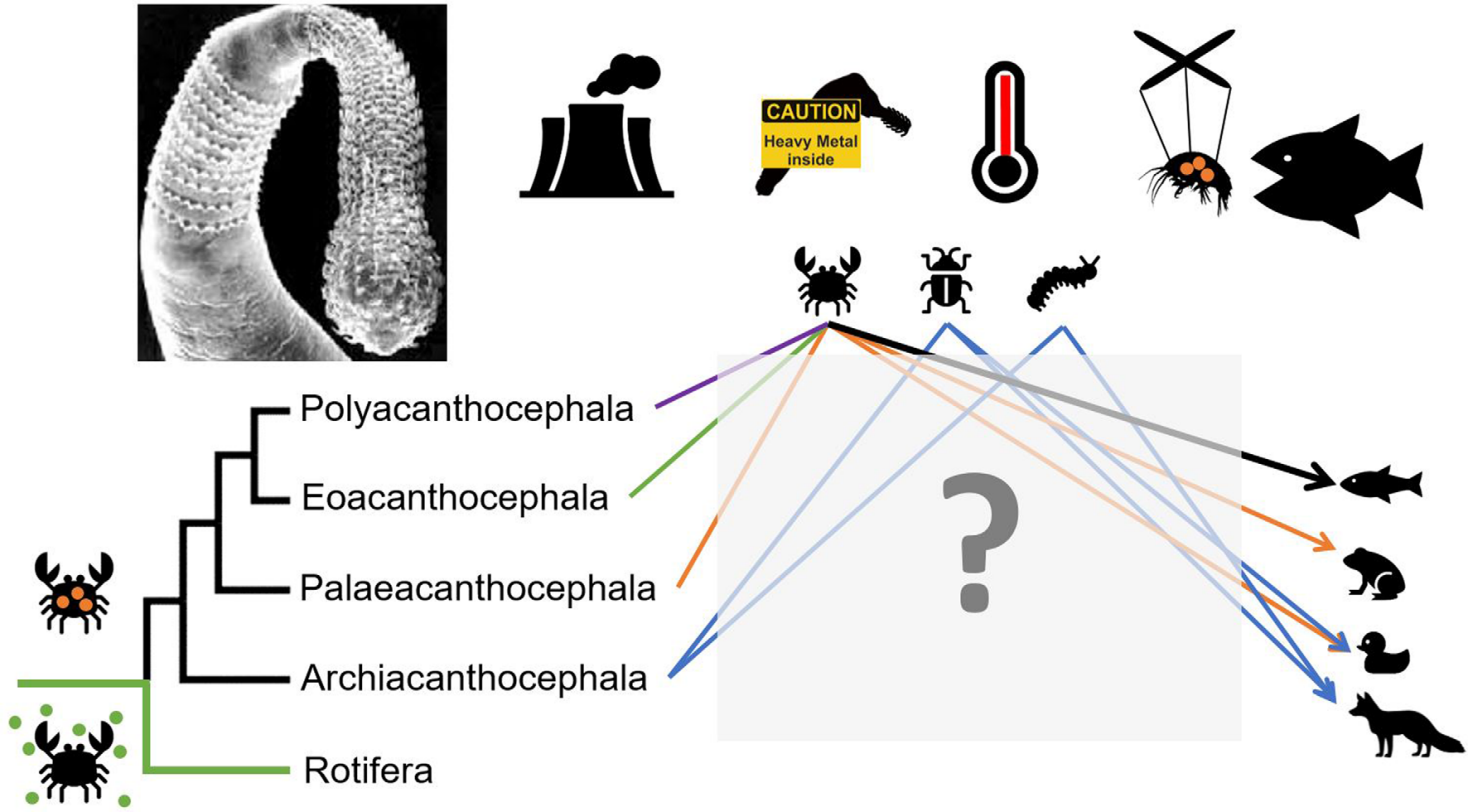
Graphical abstract of the various concepts outlined in this paper.

Acanthocephala is a small monophyletic group of heteroxenous parasites consisting exclusively of endoparasites of arthropods and vertebrates. Commonly referred to as thorny-headed worms, this group comprises almost 1300 species according to the most recent estimate published 10 years ago [6]. Long considered a separate phylum, Acanthocephala is recognized as a sister group to Seisonidea (Hemirotifera) within the Syndermata [106, 156, 158, 181, 182]. Since the first description of an acanthocephalan by Redi in 1684 and their naming by Rudolphi in 1802 [5], research on Acanthocephala has continuously fueled collective knowledge and questioning about parasite evolution and ecology. Their remarkable features stimulate the work of an international community of researchers in fields as diverse as integrative taxonomy and phylogeography, the evolution of parasitic life cycles, the ecology and physiology of host-parasite interactions, community ecology, environmental parasitology including ecotoxicology, and behavioral ecology. Part of this community meets every four years in a dedicated workshop. Following the 10th Acanthocephalan workshop held in August 2022, we wrote this opinion article to provide an insight about the knowledge gaps and a perspective on the future of acanthocephalan research. Key priorities in acanthocephalan parasites research have been categorized into three main areas: (i) Phylogeny, integrative taxonomy, and cytogenetics; (ii) Ecology and evolution of acanthocephalan life cycles, transmission, host exploitation strategies and host range; and (iii) Environmental issues related to global changes, including ecotoxicology. For each one, the most promising ideas and developments are presented based on selected case studies. Our purpose is not to cover all research gaps and future topics on acanthocephalans but rather highlight topics that are key in our opinion and/or that should attract interest from parasitologists with different expertise (Fig. 1).

Box 1 **A brief overview of Acanthocephalan origins**

Acanthocephalans are thought to have appeared during the Cambrian along with a rich and abundant marine arthropod fauna [34], switching from the free-living/epizoic lifestyle of their syndermatan ancestors to a parasitic lifestyle in aquatic arthropods [156, 182]. They subsequently evolved complex life cycles by integrating vertebrates as definitive hosts very early in their evolution. A complex life cycle is thus considered ancestral to the entire group [34, 75], with no known reversion, as predicted by the fitness benefits brought about by complex life cycle, in particular long ones [17]. Acanthocephalans probably inherited traits relevant for an endoparasitic lifestyle from their shared epizoitic (ectoparasitic?) rotifer ancestors. Among these, epidermal crypts and an invertible body apex, linked to feeding and moving habits of Hemirotifera, may have been essential for the evolution of anchoring structures and nutrient uptake in these parasites lacking an intestinal system ([156] and ref. therein). The diversification of acanthocephalan life cycles seems characterized by both constraints, mainly driven by their association with mandibulate intermediate hosts (crustaceans, myriapods and insects), and evolutionary flexibility, especially towards their definitive vertebrate hosts [118]. Acanthocephalans have colonized freshwater and terrestrial environments from marine ones, and infect the major groups of vertebrates (fish, amphibians, reptiles, birds, and mammals) [27, 67, 118]. A thorough review of the biology of Acanthocephala can be found in the eponym seminal book of Crompton and Nickol (1985) [39], which awaits an update almost 40 years later (and more than 1700 articles published on acanthocephalans since).

## Phylogeny, integrative taxonomy, and diversity

Systematic and taxonomic studies of acanthocephalans have been enhanced by the use of an expanded toolkit in the last three decades including molecular tools, as evidenced in the gradual increase in published studies integrating molecular data with morphological data since the late 1990s. However, this trend is not matched with an increase in the number of acanthocephalan taxonomists. We explain below what we think is needed to advance the scientific study of Acanthocephala systematics and evolution, by focusing on the topics of phylogenetic analyses in the area of high-throughput sequencing, and of integrative taxonomy (including non-molecular tools for advanced morphological and cytogenetic studies).

### Higher level systematics and new prospects for comparative analysis within Acanthocephala

Studying the evolution of key features of Acanthocephala including morphological traits and life cycle relies on a robust phylogeny to apply comparative methods. Acanthocephala has been morphologically classified into 4 classes: Archiacanthocephala, with four orders (Aporhynchida, Gigantorhynchida, Oligacanthorhynchida and Moniliformida), Eocanthocephala with two orders (Gyracanthocephala and Neoechinorhynchida), Palaeacanthocephala with three orders (Echinorhynchida, Polymorphida and Heteramorphida) and Polyacanthocephala with one order (Polyacanthorhynchida) [5, 6, 26]. The first molecular studies based on 18S rRNA (18S) gene sequencing and a few species representing each order supported monophyly for the three major lineages that were tested, *i.e.*, classes Archiacanthocephala, Eoacanthocephala and Palaeacanthocephala (see [61, 118]). The most recent mitogenomic studies further supported monophyly for these three major classes, but also positioned the class Polyacanthocephala within Eoacanthocephala [56, 62, 147, 181]. In addition, phylogenetic relationships remain unresolved at some order (paraphyly of Echinorhynchida), family (paraphyly of Rhadinorhynchidae) and genus levels, despite some recent efforts [56, 57, 81, 89, 114, 178, 181]. It is therefore necessary to generate larger amounts of genomic data and broader taxonomic sampling in all four major lineages to provide a robust phylogenomic framework.

In the last few decades, substantial advances in low-cost, high-throughput sequencing techniques have led to an enormous leap in phylogenomic studies of helminth parasites [82]. Currently, annotated mitogenomes of twenty-three acanthocephalan species are publicly available in which archiacanthocephalans and eoacanthocephalans remain grossly underrepresented [56]. Although mitochondrial phylogenomics offers a much higher resolution based on 12–34 genes [56, 114, 147, 181], it may also come with possible inference biases due to accelerated substitution rates and compositional heterogeneity [56, 156], and should therefore be combined with nuclear genes. The first whole genome of an acanthocephalan, *Pomphorhynchus laevis*, was generated by Mauer *et al.* (2020) [105] and was recently used to explore the phylogenomic relationships within Rotifera-Acanthocephala [106]. Draft genomes of additional species of acanthocephalans are currently being assembled [150]. High throughput phylogenomics based on Expressed Sequence Tags (EST) data [182], target enrichment of “ultraconserved elements” (UCEs) and/or Anchored Hybrid Enrichment (AHE) to design a large probe set of hundreds of loci could be implemented in the future (see Karin *et al.* [85] for a review of these phylogenomics methods). Using transcriptomic (EST) data, Wey-Fabrizius *et al.* (2014) [182] explored the evolutionary relationships within the Syndermata, in which transcriptomes of one eoacanthocephalan (*Paratenuisentis ambiguus*) and two palaeacanthocephalans (*Echinorhynchus truttae* and *P. laevis*) were included. Targeted sequence capture of UCEs has recently been applied to address schistosomatid diversification [46]. Together with extensive taxonomic sampling, high-throughput phylogenomics should provide a robust phylogeny to apply comparative methods in the future. This macroevolutionary view could shed light on host conservatism versus host switching over evolutionary time, across host taxonomic groups, and habitat [146, 180]. It will also allow for testing of evolutionary scenarios for several unique features of acanthocephalans in relation to their life cycle. More specifically, the diversification of anchoring structures and of egg and cystacanth morphology, and the evolution of phenotypic manipulation (see below), may relate to the intermediate and definitive host taxonomy, habitat, behavior and physiology [50, 128, 136, 181]. Such a comparative analysis relies on robust phylogenetic reconstruction and enhanced taxon sampling, as emphasized here.

### Integrative taxonomy and phylogeography at species-genus levels: molecular and non-molecular tools

There are now various cases in which DNA sequence data are incorporated into integrative taxonomic and phylogeographic studies of acanthocephalans at the species-genus level. Molecular data have been instrumental in investigating taxonomic status at the genus level (for instance, [89, 114, 127, 146, 180]), and in delineating species as exemplified in several cases of cryptic species or synonyms [101, 104, 127, 141, 142, 160, 162], provided that it complements thorough morphological investigation. A great example of this is the series of studies on species of the eoacanthocephalan genus *Neoechinorhynchus* Stiles and Hassall, 1905 and its relatives in Middle America [7, 58, 132–134, 145]. These studies have helped increase knowledge about diversity and variability in this unwieldy genus, but also illustrated how both morphological and molecular data must be combined to reveal the presence of cryptic species [104, 131]. The paraphyly of the genus *Neoechinorhynchus* revealed by molecular data [130] is calling for a new taxonomic revision, a rather difficult task because no compelling morphological characters have been detected yet. More generally, revisionary actions need to eventually be taken in the light of molecular data in order to bring the classification of the Acanthocephala into the 21st Century, as done for other helminth groups such as cestodes. Moving forward, we also urge researchers to use a shared and larger set of genes to maximize comparison across studies, groups, and continents for taxonomic purposes, but also to strengthen phylogenetic resolution. For example, there is a mismatch between markers used for *Neoechinorhynchus* in species studies in Asia where the 18S rRNA gene and cytochrome c oxidase subunit 1 (COI) are used [103], and in the Americas where the 28S rRNA gene and Internal Transcribed Spacer (ITS) are favored (*e.g.* [130]). Whereas 28S and ITS provided a good phylogenetic signal that enabled reliable delimitation of species and genera from diverse families of acanthocephalans [86, 145], the mitochondrial COI gene has been used to successfully define, recognize, delineate, and better understand intraspecific variation among the acanthocephalans [3, 101, 127, 131, 145, 176]. The 18S has been proven informative for taxonomic and phylogenetic studies on acanthocephalans, often in association with 28S, ITS and/or COI (more than 80 references). More recently, COI has also been used widely in phylogeographic studies with acanthocephalans of the genera *Profilicollis, Leptorhynchoides, Corynosoma, Neoechinorhynchus, Floridosentis, Southwellina, Hexaglandula,* and *Pomphorhynchus* [59, 60, 69, 127, 131, 145, 154, 160]. Therefore, all four markers classically used (18S, 28S, ITS, COI) are useful for systematic analysis on acanthocephalans, and more so when utilized all together (see *e.g.* [176]).

A variety of other, non-molecular tools have been used to study acanthocephalan taxonomy. Our recommendation is to expand the use of these complementary tools to classic morphological and molecular investigations. Scanning electron microscopy (SEM) enables parasitologists to observe detailed structures such as the proboscis, hooks, spines and pores on the surface of the body of acanthocephalans [10, 146]. Another tool, complementary to the description of proboscis ultrastructure by SEM and Transmission Electron Microscopy (TEM), is energy dispersive X-ray analysis (EDXA). EDXA provides information on part of the macroelemental composition of hooks (*e.g.*, calcium, sulfur, and phosphorus), functionally related to hook hardness *vs.* flexibility (see [8, 10]). These tools could inform on structural-functional relationships of unique features of acanthocephalans, mainly anchoring structures involved in attachment and micropores for nutrient uptake in the intestinal wall of their definitive hosts [10, 13].

Cytogenetics is another non-molecular tool that may be helpful in taxonomy. Such studies aim to characterize the organization of the genome and its changes. The karyotype is one of the basic cytogenetic characteristics of all species, and karyological data can reveal interspecific differences and similarities that are not obvious at the molecular or morphological level (*i.e.*, polyploidy, aneuploidy, different chromosomal rearrangements, and presence of supernumerary B chromosomes). Yet, the cytogenetics of Acanthocephala is still an under-researched area. The studies that have been done on this group of parasites are incomplete and focus primarily on classical karyology, *i.e.*, description of diploid chromosome number with little attention to chromosome morphology, from Giemsa-stained metaphase plates or histological sections. So far, only 12 acanthocephalan species have been karyotyped (~1% of the known species), whose chromosome numbers vary between 5 and 16 and whose sex-determining mechanisms are XX in females and X0 in males (M. Orosová, unpublished data). Due to the sparse data available for acanthocephalans, the application of karyotype data for taxonomic purpose is still very limited. However, there are some interesting examples of the usefulness of basic karyotype data in other parasite groups such as trematodes and cestodes [121, 122, 129, 161]. It is important to note that different environmental conditions may lead to intra-specific variation in karyotype, an issue that calls for careful use of this tool in taxonomic studies. For instance, B chromosomes were identified in a population of *Acanthocephalus lucii* from a heavily polluted water reservoir in eastern Slovakia, and a possible link between unfavorable environmental conditions and the occurrence of B chromosomes was suggested [159]. Complementary to karyotyping, the mapping of different repetitive DNA sequences on chromosomes by fluorescence *in situ* hybridization can provide reliable chromosomal markers that reveal some species-specific differences and could be useful in solving taxonomic and evolutionary problems also in Acanthocephala. This was demonstrated in a comparative cytogenetic study in the genus *Pomphorhynchus*, where the species-specific location of 18S loci distinguished the two sibling species *P. laevis* and *Pomphorhynchus tereticollis* [21]. Interestingly, telomeric repeats are not yet known within acanthocephalans. Fluorescence *in situ* hybridization with different telomeric probes failed to identify the chromosome end composition [22], suggesting either an as yet unknown telomeric repeat sequence or loss and replacement by other mechanisms of telomere maintenance. Genome sequencing might help in targeting this issue. In recent years, in the era of molecular phylogeny, bioinformatic analysis and genomics, it may seem that cytogenetics is an outdated technique. However, this is not the case as sequence data alone cannot provide insight into genome architecture and the role that genome architecture plays in genome plasticity [41]. Chromosomes are crucial in understanding speciation events, characterization, and often detection of cryptic species or subpopulations with specific chromosomal contents [120]. Thus, there is an urgent need to extend the limited knowledge of chromosomes in acanthocephalans. Sampling for DNA analysis is routine, now it is time to make the collection of material for karyological analysis routine as well. Finally, great care should be taken to disentangle intraspecific and interspecific variation in each of the above listed techniques in order to allow species delimitation in taxonomic studies.

### Diversity database: DNA barcode, OTU identification and good practice in vouchers deposition

Central to moving forward research on Acanthocephala is the enhancement of DNA barcode data and museum collections. Twenty years after barcoding was proposed as a basis for molecular classification of operational taxonomic units (OTUs) [138, 139], an increased effort to broaden taxonomic coverage for acanthocephalans in COI barcoding is needed to catalyze taxonomic investigation. Difficulties in OTU identification in metabarcoding approaches and ecological studies occur mostly because currently, only a minority of described acanthocephalan species has been sequenced: as for today, 116 acanthocephalan species and 186 Barcode Index Numbers (BINs) are registered in the publicly available database (BOLD system, Feb 26, 2022), *i.e.*, approximately 91% of known acanthocephalan species are not yet barcoded. In addition, the development of a comprehensive database of reference sequences, derived from accurate identifications of vouchers based on morphological grounds, is critical for OTU identification in metabarcoding approaches. It is complementary to the deposition of type, morphological and molecular voucher specimens in recognized, permanent and publicly accessible repositories, concomitantly to local repositories. This practice of “biobanking” [12] has steadily increased in recent years, with researchers depositing hologenophores (*i.e.*, the acanthocephalan specimen from which molecular data was generated) or paragenophores (*i.e.*, conspecific specimen vouchers collected from the same hosts, at the same time and locality as the molecular specimen) in a number of international repositories. Unfortunately, the deposition of additional molecular vouchers (*i.e.*, whole worms, or parts thereof, preserved in 95–100% ethanol) to natural history collections that possess biobank repositories is not standard practice among acanthocephalan researchers. Therefore, we strongly advocate the use and deposition of morphological vouchers of molecular specimens and cross-referenced molecular vouchers as common practice in future acanthocephalan molecular studies, to ensure future taxonomic or ecological studies and the generation of new molecular data, including DNA barcodes.

Box 2 **-omics tools, phylogeny and integrative taxonomy in acanthocephalans: past and future**

- High-throughput phylogenomics and broader taxonomic coverage should now be considered to provide robust phylogenetic relationships in Acanthocephala and a reliable phylogenetic framework for the comparative analysis of acanthocephalan features in relation to endoparasitic lifestyle and host diversity.
- Modern -omics approaches should generate novel data on the molecular mechanisms involved in their adaptation to both invertebrate and vertebrate hosts from their syndermatan ancestors, in particular anchoring structures, nutrient acquisition strategy and immune evasion mechanisms.
- Integrative taxonomy so far relied on COI, ITS and 28S, 18S genes. Optimal synergy of molecular and morphological data, as well as non-molecular tools, will help to clarify species delimitation and to support taxonomic diagnosis.
- Barcoding and cytogenetics are lagging, with only 9% and 1% of species analyzed, respectively. It limits modern taxonomic investigation based on cross-check of morphological identification through barcode analysis, and the exploration of existing issues such as karyotype evolution, gene duplication/inversion, and telomere evolution.
- Strengthening the infrastructure of this research on Acanthocephala requires an open access database and the systematic deposition of molecular and morphological vouchers in curated museums, and an increased effort in barcoding.

## Ecology and evolution of acanthocephalan life cycles, exploitation and transmission strategy, and host range: filling knowledge gaps by taking multidisciplinary approaches

Acanthocephalan life cycles often include several possible intermediate and definitive hosts [27, 67, 87]. Both host availability and parasite plasticity in host exploitation shape life cycle, host range and transmission strategy of acanthocephalans at ecological and evolutionary timescales. Their dynamic changes more specifically include the frequency of occurrence of host switching or host acquisition, of paratenic host incorporation or of post-cyclic transmission (*i.e.*, “When ingested as adults within their definitive hosts some acanthocephalans survive and parasitise the predator” [119]). Documenting these changes allows us to address the associated changes in virulence towards intermediate and definitive hosts, in parasite development and reproduction, and in host manipulation. It thereby allows for testing of general predictions about the costs and benefits in evolving complex life cycles [17]. We underscore here three topics with particularly notable knowledge gaps in the life cycle and host exploitation strategy of acanthocephalans (i) the incomplete resolution of life cycles, mainly due to a lack of records of intermediate hosts; (ii) the fragmentary understanding of host manipulation by acanthocephalans as part of their transmission and host exploitation strategies (physiology, immunity), based on a small number of species and only occasional interest in the underlying mechanisms; and (iii) the understudied factors contributing to plasticity in host range and specialization, such as ecological opportunities brought about by biological invasion and changes in trophic network.

### Life cycle of acanthocephalans: fragmentary knowledge

Knowledge of acanthocephalan life cycles has historically been growing thanks to parasitological surveys of macroparasites (helminths) on one or few focal definitive host species, mainly fish, waterbirds, amphibians, and reptiles. Their ecology has been thoroughly reviewed by Kennedy, 2006 [87]. Yet, as for most parasitic groups, this knowledge is still fragmentary. In fact, despite an increasing number of species descriptions in various clades of acanthocephalans, in most cases, intermediate hosts are often unknown or only inferred from knowledge of closely related parasite species (*e.g.* [9, 37, 68, 157]), especially for marine acanthocephalans [27]. Elucidating life cycles is difficult because larval and juvenile stages of acanthocephalans (acanthella, cystacanth) are hardly detectable and/or difficult to identify using morphology, and because of low prevalence of infection in intermediate hosts. This knowledge gap is unfortunate for trophic-network ecologists in particular [20], for whom understanding each species-species interaction is critical [113]. Since acanthocephalan life cycles involve trophic transmission and often include several intermediate/paratenic hosts, any gap in knowledge of host-parasite relationship will indeed lead to a misunderstanding of trophic networks. For example, the well-studied *P. laevis* was seen as a parasite without paratenic hosts [87] until the discovery of high prevalence of cystacanths in the body cavity of minnows (*Phoxinus phoxinus*) and gudgeons (*Gobio gobio*) [111]. Since these extra-intestinal cystacanths are then infectious for chubs (*Squalius cephalus*; Sures, pers. comm.), a well-established definitive host, minnows (and presumably gudgeons) are paratenic hosts in the *P. laevis* life cycle [111], revealing new nodes in the trophic networks in European rivers. Other studies have been dedicated to finding new host species by using morphological techniques (*e.g.*, [140]), but such studies are time-consuming and sometimes not fully reliable.

Molecular techniques can reduce search times and improve the accuracy of finding intermediate or paratenic hosts. For instance, DNA barcoding can be used to link larval stages of acanthocephalans in intermediate hosts to adults in definitive hosts, and to confirm or rule out the role of target species as intermediate hosts [3, 90]. The detection of acanthocephalans in various habitats or hosts could also rely on environmental DNA (eDNA) metabarcoding. This latter technique opens practical, fast and sensitive avenues to characterize parasite diversity from specific hosts or in a locality by screening eDNA samples, *i.e.*, from soil, sediment, filtered water, feces, tissue, *etc.* [14]. It has already generated novel information for biodiversity assessment of helminth parasites [45, 148] and new host-parasite associations. Acanthocephalan-like OTUs have already been detected in metabarcoding screening of gut and fecal samples from wild hosts. For example, de Vos *et al.* [179] detected acanthocephalans (most probably belonging to *Bolbosoma*) in fecal samples of blue whales from the Northern Indian Ocean using a dietary DNA metabarcoding approach. Later, Elsaied *et al.* [47] recorded a *Neoechinorhynchus-*like OTU in the gut of Nile tilapia (*Oreochromis niloticus*) from Lake Nasser, Egypt. Recently, Verkuil *et al.* [177] detected unidentified acanthocephalans in fecal samples of Pied Flycatcher (*Ficedula hypoleuca*) from the Netherlands. Metabarcoding could therefore be used as a monitoring tool of acanthocephalans in a wide range of habitats, to detect new acanthocephalan–host associations, while reducing the need for complex host necropsies for monitoring purposes and for well trained taxonomists. However, it calls for an increased effort to generate DNA barcode data and identification in acanthocephalans, because currently only a minority of described acanthocephalan species has been sequenced (see above).

Obtaining new data on the intermediate hosts of acanthocephalans (more generally on their life cycle) is one thing, but sharing this knowledge in an open way is another. There is a need for an open database including information on host range (definitive, intermediate or paratenic), but also morphological and molecular information (see above), for several reasons. First, it would make it easier to share this knowledge in the community of researchers on acanthocephalans. Second, at an applied level, parasites can have a significant impact on commercial aquaculture, by lowering growth, reproduction and survival rates of infected hosts [155]. Therefore, any advance in understanding the life cycle of parasites can help control them. Third, this kind of information is important for trophic-network ecologists. Such a database should be part of or connected to the diversity database (see above) to collate information on DNA barcodes (BINs), OTUs and morphological characteristics, with ecological features including host range (definitive, intermediate or paratenic) and habitat. The data already available in books (*e.g.*, [87]), review papers [27, 67], or in existing databases [16] should be transferred to an open online database and updated. This should also facilitate the identification, correction and communication of acanthocephalan species misidentifications as exemplified in the case of *P. tereticollis* and *P. laevis* confusion (see also Reier *et al.*, 2020 [142] for another example). Thanks to COI and ITS identification of *Pomphorhynchus* sp. and the use of eDNA to detect and identify amphipod species, *P. tereticollis* was shown to be the species present in rivers in the United Kingdom and infecting *Gammarus fossarum*, while it was thought to be *P. laevis* and *Gammarus pulex* for over 70 years [18, 73, 127]. This confusion extended to definitive hosts: fish reported as definitive hosts of two “strains’’ of *P. laevis* in the UK [76, 77, 88] were actually infected with *P. tereticollis* and were in fact species known to be the preferred hosts of *P. tereticollis* in continental Europe [11, 124]. The value of a database to centralize important updates on host range and/or acanthocephalan identifications (or misidentifications) is also exemplified for freshwater fish in North America. Since the major treatise by Hoffman, the 2nd edition of which was published in 1999 [78], there have been many changes to species identifications and subsequent works on genera such as *Neoechinorhynchus,* as mentioned above, but there has been no centralized document or resource to publish these changes [152], which should be done in the future.

### Addressing flexibility in life cycle and host specificity in the context of global changes

Flexibility in life cycle and the associated changes in host range can be addressed using cases of species introductions and climate change, two major causes of species range expansion. Alien host and acanthocephalan co-introductions can lead to local host capture by alien acanthocephalan species (spillover effect) or introduced host capture by local acanthocephalans and resulting spillback effect on the local host. A co-introduction process occurs when a host-parasite pair is introduced into a new geographic area. This phenomenon is not less likely to be successful in heteroxenous parasites compared to monoxenous ones, despite the constraint of using at least two obligate hosts [17, 102]. Acanthocephala is a potentially useful model group: many species are aquatic (marine or continental) and use crustaceans as intermediate hosts, two features associated with a high rate of biological invasions [63, 74, 94]. The case of co-introduction in the Danube and Rhine River systems of a *Pomphorhynchus* species with the Ponto-Caspian round goby *Neogobius melanostomus*, is an excellent case-study [40, 48, 53, 79, 141]. The invasive goby acts as a paratenic host in the invaded area where local fish species (the European barbel, *Barbus barbus* and the chub, *Squalius cephalus*) act as definitive hosts. The prior introduction and establishment in the Danube and Rhine River of its amphipod intermediate host, *Dikerogammarus villosus*, has probably initiated and facilitated the co-introduction of *P. laevis* (*bosniacus*) [79].

A second research avenue consists in monitoring the response of acanthocephalans to climate change, using a longitudinal study design to monitor the changes in host range, parasite transmission, and basic epidemiological parameters (acanthocephalan prevalence, intensity, fecundity, *etc.*). Climate-induced changes in host-parasite interactions are manifold. Changes in temperature can alter acanthocephalan development times [91, 174], and acidification and dissolved oxygen concentrations could also impact parasite transmission, by altering intermediate host behavior [183]. Combined with the effects of warming and hypoxia on invertebrate immunity [31, 32, 80, 95], climate change can therefore deeply affect the acanthocephalan life cycle, epidemiology, and local host range. Addressing these issues is probably very challenging considering the complexity of individual-, population- and community-level responses to climate change [91]. This has already been advocated in a review on the consequences of Arctic warming on the diversity, circulation and transmission of helminths in Arctic coastal ecosystems [55]. Such a challenge will require cooperation among parasitologists involved in long-term and ecosystem-specific parasitological monitoring.

### Host exploitation strategies: immunity and nutrient uptake

At the level of host-parasite interactions, host exploitation and transmission strategies have been the focus of intensive research these past 70 years (reviewed in [38, 39, 50, 172]), but based on a handful of acanthocephalan species. Despite numerous studies thoroughly reviewed by Crompton (1970) [38], Schmidt (1985) [149], and Taraschewski (2000) [172], host exploitation strategies by acanthocephalans still raise puzzling questions, particularly regarding immune evasion and energy uptake and allocation, in both intermediate and definitive hosts.

Circumventing or depressing host immune defense is as vital as nutrient uptake to any endoparasite. In the intermediate host, the early step of development involves the acquisition of a “capsule” or “acellular envelop” by the acanthocephalan larvae. The function and origin of this outer envelope were the subject of studies in the 1960s to 1990s (reviewed by Crompton (1970) [38], Schmidt (1985) [149] and Taraschewski (2000) [172]). Its function is probably to evade the recognition mechanism of the host, as evidenced in the lack of healthy hemocyte accumulation on developing acanthellae and on cystacanths. Such protection relies on yet unidentified properties of this outer envelope, possibly based on the acquisition and incorporation of host proteins thereby masking parasite antigens [172]. However, this protective function has not been unambiguously established yet and certainly warrants further investigation. In addition, there is still controversy on the origin of this outer envelope [43, 172], that may be resolved by considering temporal alterations in its constitution. Made initially from the connective tissue surrounding the intestine upon penetration of the acanthor into the arthropod hemocoel, it may be progressively replaced during acanthella development by membranous material of parasite origin together with residues of disintegrated hemocytes [172], a hypothesis that needs further testing. The alteration of host immune system by acanthocephalan acanthella and cystacanth also leads to immunosuppression in the intermediate host (reviewed in [50, 172]). Immunosuppression in the few host-acanthocephalan species investigated was evidenced by lower humoral immunity and cellular immunity at the cystacanth stage (prophenoloxidase system and hemocyte concentration respectively [35, 143], reviewed in [50] and [172]). The proximate mechanisms underlying these alterations have not yet been studied, and the fitness outcome has barely ever been estimated. The molecular triggers and the timing of immunosuppression during larval development in the intermediate host are not yet known, but likely involve acanthocephalan excretory–secretory products that impair or inhibit both cellular and humoral effectors of the intermediate host immune system. In terms of fitness outcome, the evolutionary significance of immunosuppression in the intermediate host is not clear [36, 50]. Indeed, while immunosuppression may benefit the parasite by facilitating immune evasion and/or by saving host energy for its own growth/survival, it may also compromise the survival of infected hosts in response to secondary infection with other pathogens [35, 36]. Lastly, the neuropsychoimmune hypothesis of manipulation, suggesting a functional link between immunosuppression of the intermediate host and behavioral manipulation [1] (see below), is still left unexplored. At the adult stage, infection with acanthocephalan elicit a well characterized inflammatory response in the intestinal tract of fish definitive hosts (reviewed in Dezfuli *et al.* (2016) [42]). Histopathological observations of fish intestines revealed local response at the point of parasite attachment, with mucous cell hyperplasia and hypertrophy, and the accumulation of immune cells in response to mechanical damage and sometimes a fibrous capsule of inflammation tissue around the deeply penetrating proboscis [42]. An interesting perspective would be to address the link between intestinal inflammation and the dysbiosis recently evidenced in fish infected with *P. laevis* [33], since the alteration of gut microbiota may subsequently affect host metabolism and intestinal immune system, and ultimately host fitness.

As for parasite development and immune defense, nutrient uptake was the focus of active research in the 1960s to 1990s, and renewed interest in this topic has been brought about by new tools, more specifically isotope studies and transcriptomic studies. Most of these studies focused on definitive hosts. Using stable isotope analysis of carbon and nitrogen as a tool to unravel trophic relationships, it emerged that adult acanthocephalans are depleted in the heavier nitrogen isotope ^15^N, which shows that they feed mainly on metabolites provided by the host [84, 115]. The pattern of reduced δ^15^N (ratio of ^15^N/^14^N) when compared to definitive host tissues can also be found in cestodes [15, 64] but is in contrast to nematodes for example [115]. Accordingly, there are clear differences with respect to the trophic interaction between different parasite taxa and their hosts. Taxa actively feeding on host tissues, such as adult nematodes and monogeneans [64], can be clearly differentiated from acanthocephalans and cestodes that behave as absorptive feeders. Transcriptomic analyses in adult acanthocephalans revealed several genes involved in energy metabolism and carotenoid uptake especially under anaerobic conditions (fermentation) [105, 151]. Importantly, definitive host response to intestinal acanthocephalans involves coordinate responses from the enteric neural, endocrine, and immune systems (see Bosi *et al.* (2022) [23] for a review in fish), which relies on molecular crosstalk between host and parasite molecular effectors and modulators. Detailed transcriptomics and proteomics analyses should be encouraged in the future in a broader range of acanthocephalan species, to unravel the physiological/molecular pathways of nutrient acquisition in adult acanthocephalans and the molecular basis of inflammation, but also the impact of infection on gut microbiota [33], parasite resource acquisition and allocation in optimal and sub-optimal hosts [151], and ultimately the fitness for both the acanthocephalan and its host. This should also contribute to understand the evolution of acanthocephalan virulence towards their definitive hosts, as under some environmental conditions, acanthocephalans might actually be beneficial to their definitive hosts (*cf.* third section below).

### Manipulation as an adaptive transmission strategy: opening new paths

Parasites with a complex life cycle are under strong selection pressure to evolve transmission strategies that bridge the gap between successive obligate hosts, one of which is intermediate host manipulation [17, 50]. Acanthocephalans are excellent model organisms for the study of intermediate host manipulation ([50] and references therein). The effects of acanthocephalans on their intermediate hosts’ behaviors, life-history, morphology, and physiology (reviewed in [39, 50, 87]) may make them more likely to increase their chances to be transmitted to their definitive host. In their meta-analysis, Fayard *et al.* [50] confirmed that larval stages of acanthocephalans generally induce significant changes in taxis (increased habitat overlap with predator), responses to predator stimuli, immunity (immunosuppression), reproduction (castration) and coloration (increased conspicuousness), in their intermediate hosts. Most importantly, predation by suitable definitive hosts is enhanced during the cystacanth (mature, transmissible) stage of at least five palaeacanthocephalan species, providing further evidence that these phenotypic alterations are adaptive for the parasite, which is rarely demonstrated in other groups of putatively manipulating parasites [137]. However, this knowledge is still fragmentary, mainly due to two biases or gaps in research: species coverage and proximate mechanisms.

First, there is a general bias in the study of phenotypic manipulation towards a handful of acanthocephalan species. This considerably limits the comparative analysis of manipulation across the taxa [50]. The issue at stake is the relative importance of convergence versus homology in the evolution of manipulation. Under the evolutionary convergence hypothesis, ecological constraints (mainly definitive host habitat use and diet) led to similar strategies of intermediate host manipulation. Under the homology hypothesis, similar host exploitation strategies, including manipulation, reflect shared ancestry and likely constrain host switching/acquisition. This chicken and egg conundrum was raised some time ago [29], but remains to be addressed. A broader identification of acanthocephalan intermediate hosts and accurate characterization of phenotypic alterations – or even better, of their underlying molecular mechanisms – followed by ancestral state reconstruction along a robust phylogeny, represents a colossal task. However, it could allow us to decipher which manipulation strategies are convergences and when the homologous ones originated.

Second, knowledge of the mechanisms underlying behavioral alterations in intermediate hosts is still in its infancy, and mainly restricted to candidate neuromodulatory pathways. For instance, the serotonergic pathway is likely involved in behavioral alterations induced by *P. laevis* in the amphipod host *G. pulex* [126, 170, 171]. In *Gammarus roeseli* individuals infected by the bird parasite *Polymorphus minutus*, negative geotaxis may be triggered by infection-induced hypoxia or hypoxia-signaling, as suggested by the reversal of geotaxis in uninfected individuals injected with lactate, and in the elevated brain lactate concentration in *P. minutus*-infected individuals compared to uninfected ones [125]. In these studies, changes in taxis, and thereby habitat preferences, were mimicked by the pharmacological manipulation of candidate (neuro-) physiological pathways. The corresponding alteration in brain chemistry (serotonin, lactate) of infected gammarids confirmed the involvement of these pathways. In addition, the striking and efficient ways for acanthocephalans to get transmitted is not only to change habitat preferences but also to alter anti-predatory behaviors. Therefore, any study on the proximate mechanisms of manipulation leading to increased acanthocephalan transmission to definitive hosts should investigate pathways modulating antipredator behavior.

These studies addressed the mechanisms underlying manipulation by acanthocephalans by targeting candidate neuromodulatory or physiological systems of the host. The prospects and limitations of such approaches have been reviewed, highlighting the complexity of these systems and the fact that they still show a host’s physiological response to manipulative parasites rather than evidencing the actual nature and targets of parasite excretion-secretion products [123]. In future studies, two complementary approaches may be taken: the proteomic analysis of excretion-secretion products of acanthocephalan larval stages, as done in trematode species [70, 72], and the quantification of gene expression in both the intermediate host brain and the larval stage of the acanthocephalan. This could enable screening for candidate proteins involved in host-parasite crosstalk, providing insights into the exact pathways involved, and identifying parasite genes that may be under natural selection for manipulation. Whole genome and transcriptome sequencing of *P. laevis* adults [105] provided a very useful tool to tackle this quest, which needs to be reproduced at the acanthella and cystacanth stages. In this perspective, the fact that acanthocephalans seem to follow the switcher paradigm [83] provides an original and unique opportunity in the study of host manipulation by trophically-transmitted parasites. The switcher paradigm proposes that manipulative parasites first decrease predation risk of the infected intermediate host at larval stages not yet infective to definite hosts, and then enhance predation by definitive hosts when the parasite larva is infective ([44]; reviewed in [50]). The comparison of acanthellae and cystacanths transcriptomes combined to extended phenotype characterization would give the opportunity to pinpoint potential genes - or their pleiotropic regulation linked to behavioral and immune alteration induced in the intermediate host - thereby deciphering the molecular basis of manipulation switch.

Box 3 **Addressing the complexity of acanthocephalan life cycles**

- Intermediate hosts of most acanthocephalan species are unknown, which biases our understanding of trophic networks. DNA (meta-)barcoding has the potential to facilitate the detection of parasites in environmental DNA and the identification of larval stages in intermediate hosts.
- Variable flexibility in acanthocephalan life cycle provides opportunities to test hypotheses regarding the effect of co-introductions of alien host-parasite pairs on local communities.
- Climate changes alter host-parasite interactions, with likely cascading effects at population and community levels, especially as these parasites affect predator-prey interactions.
- Parasitologists need an online database gathering morphological descriptions, molecular data, and ecological information such as geographic distributions and host ranges of thorny-headed worms.
- An integrative approach to the immediate mechanisms of alterations in host energy metabolism and immune response in intermediate and definitive host is needed, relying on proteomic analysis of excretion-secretion products and transcriptomic analysis at different stages of parasite development.
- Although excellent model organisms for the study of host behavioral manipulation, mechanisms thereof were rarely investigated and are far from elucidated, including the link between immune evasion/immunosuppression and behavioral manipulation.

## Environmental aspects – interactions between acanthocephalans and pollutants

Given the complex interactions between infection with parasites and exposure to pollutants, environmental parasitology has developed as a discipline focusing on the potential role of parasites as indicators of environmental health as well as on the combined effects of parasites and pollutants on the health of the hosts [153, 166]. Acanthocephalans have been widely recognized as potential indicators of pollutant accumulation [167], and these parasites might lead to host detoxification and subsequent (beneficial) effects on host health or physiology [112]. The mechanisms involved in host detoxification processes, as well as the effects of pollutants on development and transmission of acanthocephalans, are largely unknown and should therefore be of increased research interest in the future.

### Acanthocephalans as accumulation indicators of pollutants

Acanthocephalans are able to accumulate a variety of metals at higher levels than their hosts (reviewed in [116, 165, 166]). Even though metals are not detected in the host or in established free-living bioindicators such as bivalves, evaluation of metal occurrence in the environment is still facilitated by metal accumulation in acanthocephalans in their definitive hosts [167, 168]. A similar pattern was reported for polychlorinated biphenyl (PCB) accumulation in a perch-acanthocephalan system [24]. Metabolizable organic compounds such as phthalates and polycyclic aromatic hydrocarbons (PAHs) are also accumulated in the acanthocephalan at higher concentrations than in the chub host [112]. This observation might be due to an ability of the acanthocephalan *Pomphorhynchus* sp. to metabolize organic compounds or to take up metabolites formed in the host [112].

Moreover, the presence of contaminants in acanthocephalans is evidence not only of the presence of substances in the host’s environment, but also of their biological availability in general [166]. The substances detected in the body of acanthocephalans must be considered biologically available because they had to pass through the teguments and membranes of the parasite, since acanthocephalans lack a digestive tract. This unique substance uptake mechanism, which can be found not only in acanthocephalans but also in cestodes, makes such gutless taxa promising indicators for studies on the bioavailability of (nano)particles. If particulate substances or the elements that form them can be detected in Acanthocephala, as shown for particulate platinum-group metals [164, 184], they must pass through several biological membranes and are thus bioavailable. On the other hand, if filter-feeding organisms such as bivalves are used to study the uptake of (nano)particles, it remains unclear whether these elements are only adsorbed on the gill filaments or are present in the gut contents without having been taken up in a biological sense. Acanthocephalans could help to fill this gap and provide a better understanding of the biological availability of (nano)particles and other pollutants in ecosystems. In addition to metallic nanoparticles, this could also be a promising approach to study the uptake, accumulation, and effects of nano- and microplastic particles in the aquatic environment.

Recently, acanthocephalans have been included in physiologically-based toxicokinetic models simulating metal accumulation in fish-acanthocephalan systems [96, 97], which are based on the conceptual model developed by Sures *et al.* (1999) [168]. In these models, acanthocephalans are considered a sink for metals in the host intestine, thereby reducing metal availability to fish organs [167, 168]. These models provide a mechanistic explanation on the substantial variations in the partitioning of metals in fish-acanthocephalan systems [51, 52, 117, 135, 167, 173]. Accordingly, the variations in the accumulation potential in acanthocephalans among metals are not attributed to any metal-specific uptake mechanisms, but to the availability of the metals in the host intestine [97]. In particular, the accumulation of metals in acanthocephalans depends on metal-specific organotropism and cellular fate [97]. Similar models for the accumulation of organic contaminants by acanthocephalans [98] are currently lacking but would be highly desirable.

### Effects of acanthocephalans on physiological responses of the host

The accumulation of pollutants in the host might lead to various physiological responses at different organization levels, from the synthesis of stress molecules and protective enzymes to a disruption of physiological homeostasis and mortality. Most research performed so far on combined effects of pollutants and acanthocephalans refers to larval acanthocephalans mainly in their intermediate crustacean hosts [71]. This is interesting in that larval stages are not able to accumulate contaminants to the same degree as adult Acanthocephala [169] and therefore do not generally lead to lower contaminant concentrations in infected intermediate hosts compared to uninfected conspecifics. However, infection-inhibited accumulation of metals in gammarids has been described (although metal accumulation in the cystacanths was much lower compared to the gammarid host), which might reduce adverse effects on the host [66]. Obviously, the mechanisms leading to altered metal accumulation in intermediate versus definitive hosts are completely different and deserve further mechanistic studies.

Also, the effects of concurrent contamination and infection on the physiology of the corresponding intermediate hosts show wide variations. For example, the acute toxicity of Cd to *G. pulex* was not affected by infection with the acanthocephalan *P. laevis* [108]. The results of several other studies showed that twice as many amphipods infected with cystacanths of *P. laevis* or *P. minutus* died compared to uninfected conspecifics when exposed to low metal concentrations [54, 107–109]. In contrast, in the study of Sures and Radszuweit (2007) [163], the mortality of *P. minutus*-infected *G. roeseli* was reduced following Pd exposure compared to uninfected individuals. Similar observations with the same parasite−host system during Cd exposure were made by Gismondi *et al.* (2012) [66] for the mortality of infected male gammarids. Such contradictory findings may be the result of complex combined effects of acanthocephalan infection and exposure to contaminants on host physiological homeostasis, which could be modulated primarily by infection with acanthocephalans. The mechanisms responsible for the different survival rates of infected and uninfected gammarids in the presence of concomitant exposure are completely unknown and therefore represent an exciting area of future research.

Various results on the influence of exposure to pollutants on antitoxic responses have been reported. Stress proteins (also termed “heat shock proteins”) are synthesized by organisms in response to different environmental stressors such as exposure to metals, organic pollutants, ultraviolet radiation, temperature changes, and osmolarity or hypoxia/anoxia [100]. The induction of heat shock proteins in the gammarids *G. roeseli* and *G. fossarum* in response to metal (Pd and Cd) exposure was modulated by the infection with cystacanths of the acanthocephalan *P. minutus* [54, 163]. Such modulation of the hosts’ repair and detoxification systems might enhance adverse effects of environmental pollution on the hosts. Similarly, the synthesis of metallothioneins, a specific biomarker for metal exposure [4], in the gammarids was enhanced by the infection with acanthocephalans, possibly *via* the influence on the transcription of the metallothionein gene [65]. However, such infection enhanced cell damage as evidenced by an increase in malondialdehyde concentration, a product of metal-induced lipoperoxidation, in the gammarids *G. roeseli* [65]. In summary, infection with larval acanthocephalans lowers the efficiency of the overall defense system in gammarids exposed to metals [65]. Another explanation for the more severe cell damage in the infected gammarids could be a high energy investment in antioxidant defense and in the parasite’s metabolism [65], which was supported by substantial decreases in lipid and glycogen contents [30, 65]. Infection with acanthocephalans might affect metal tolerance of the host also *via* other mechanisms. For example, increased sensitivity of gammarids infected with cystacanths to Cd was assumed to result from parasite-induced alterations of the host’s feeding rates (*e.g.*, [25, 110]).

Compared to the combined pollutant and parasite effects on the intermediate host, information on their effects on definitive hosts is rather limited. However, interesting results have recently been published. Infection with the acanthocephalan *Tenuisentis niloticus* stimulated the activities of reduced glutathione and catalase in the fish host [2]. Another example is the lower oxidative stress and damage induced by emerging organic pollutants (*e.g.* PAHs, PBs, OCPs, PBDE, DEET) in acanthocephalan-infected chubs in comparison with that in uninfected conspecifics [112]. In addition to such effects on the molecular responses to pollutants, infection with parasites might affect the microbial community in the definitive host [33]. In particular, the European chub infected by *Pomphorhynchus* sp. hosted fewer bacterial communities than uninfected conspecifics [33]. These examples demonstrate the relevance of interactive effects between pollutants and adult acanthocephalans, which also seems to be a valuable area for future research activities. Moreover, the relationship between acanthocephalan-inhibited oxidative stress and the acanthocephalan-inhibited internal concentration of pollutants in the definitive host is still not addressed. An understanding of this link might provide us with a mechanistic understanding on the combined effects of pollutants and acanthocephalans on the definitive host.

### Effects of environmental stressors on development and transmission of acanthocephalans

Compared with what we know about the influence of Acanthocephala on pollutant accumulation, our knowledge about the effects of environmental pollution on acanthocephalan development is largely limited and results are conflicting. Lewis *et al.* (2003) [99] suggested that acanthocephalans can respond quickly to environmental changes. For example, the abundance of the acanthocephalan *Pandosentis* aff. *iracundus* was negatively correlated with the concentrations of ammonia and dissolved oxygen [92]. However, the influence of dissolved oxygen concentration should be carefully interpreted because of: (i) the correlation between oxygen concentration and water temperature; and (ii) the impact of water temperature on acanthocephalan reproduction [87]. Similarly, acanthocephalans might be adversely affected by metals [93]. Other studies reported a higher abundance of acanthocephalans at sites with higher organic/nutrient loads and eutrophication levels, respectively (see *e.g.*, [49, 175], which was assumed to be linked to a higher pollution tolerance of intermediate and definitive hosts. Accordingly, acanthocephalans might be directly affected by pollutants or indirectly *via* impacts on the host [28]. A correlation between the occurrence of acanthocephalan parasites and the abundance of intermediate hosts unraveled by Fanton *et al.* (2022) [49], is in contrast to a study that reported no significant effects of environmental stressors, such as pollution on acanthocephalans [19]. Such contradictory findings suggest a lack of thorough understanding of the effects of environmental stressors on the development and transmission of acanthocephalans. Therefore, future research needs to address the mechanisms of action for single acanthocephalan species, also considering their host specificity and the preferences of their hosts for the environmental conditions.

Box 4 **Acanthocephalans at the forefront of research in environmental parasitology**

- Detailed studies on the uptake, accumulation, and potential effects of organic pollutants on acanthocephalan-host relationships should complement current knowledge.
- Acanthocephalans could serve as indicators of particulate pollutants such as microplastics and nanoparticles.
- Interdisciplinary and mechanistic studies elucidating processes of host detoxification by acanthocephalans as wells as their potentially beneficial effects on the physiology and health of their definitive hosts are highly desired.
- Omics approaches should be used to obtain information on the effects of pollutants on parasites.

## Conclusion

Research on the Acanthocephala illustrates the variety of topics of investigation arising from their unique features. To frame this research into a robust phylogenetic/phylogenomic framework, the taxonomic and genetic coverage of current phylogenies should be broadened. It would contribute not only to the systematic resolution of this group, but also to the study of the evolution of life cycles (transitions between marine, freshwater and terrestrial environments, type and diversity of intermediate and definitive hosts, patterns of phenotypic manipulation, *etc.*), as well as the evolution of genome architecture (repeated sequences, B chromosomes, karyotypes, and telomere maintenance). For most of these issues, data are still scarce and often restricted to a handful of species, as evidenced in the fragmentary knowledge about acanthocephalan cytogenetics, life cycles, or phenotypic manipulation. Yet, we hope that acanthocephalan research will continue to be an active area in the future and attract researchers from other fields. The study of acanthocephalans has long been undertaken by expert scientists with diversified skills, scientific questioning, and approaches. Most of them have interests in other parasite groups (noticeably helminths) and/or in the ecology/evolution of their hosts (mainly fish and crustaceans), thereby nurturing multidisciplinary research. However, the perennial challenges that remain are the low number of experts and the perception that this group has little impact on human and domestic animal health. These challenges have limited the extent of scientific input and the scope of this research compared to other parasite taxonomic groups.

Parasites are now perceived as important components of ecosystems, with impacts on trophic networks and nutrient flows, and have an acknowledged value as bioindicators of anthropogenic perturbations. Considering the present-day issues of biodiversity conservation and ecosystem stability/resilience in the face of anthropogenic changes, research on acanthocephalans should not lag behind because of a lack of appeal and funding. We hope to have convinced our readers of the excellent opportunities provided by acanthocephalans as a model system to many fundamental and applied aspects of the evolution and ecology of host-parasite interactions. Tools necessary to collectively improve our effectiveness and visibility include the enhancement of cross-referenced molecular voucher specimens in museum collections together with morphological vouchers, and the creation of an online open database gathering molecular barcoding, morphospecies and ecological (life-history and life cycle) data. This will contribute to promoting and coordinating research efforts in integrative taxonomy and in the ecology and evolution of the Acanthocephala.
